# Trauma-related guilt as a mediator between post-traumatic stress disorder and suicidal ideation

**DOI:** 10.3389/fpsyt.2023.1131733

**Published:** 2023-03-28

**Authors:** Po-Han Chou, Shao-Cheng Wang, Chi-Shin Wu, Masaya Ito

**Affiliations:** ^1^Department of Psychiatry, China Medical University Hsinchu Hospital, China Medical University, Taichung, Taiwan; ^2^Department of Psychiatry, China Medical University Hospital, China Medical University, Taichung, Taiwan; ^3^Department of Psychiatry, Taoyuan General Hospital, Ministry of Health and Welfare, Taoyuan, Taiwan; ^4^Department of Mental Health, Johns Hopkins Bloomberg School of Public Health, Baltimore, MD, United States; ^5^Department of Medical Laboratory Science and Biotechnology, Chung Hwa University of Medical Technology, Tainan, Taiwan; ^6^Department of Nurse-Midwifery and Women Health, National Taipei University of Nursing and Health Sciences, Taipei, Taiwan; ^7^National Center for Geriatrics and Welfare Research, National Health Research Institutes, Miaoli, Taiwan; ^8^Department of Psychiatry, National Taiwan University Hospital Yunlin Branch, Yunlin, Taiwan; ^9^National Center for Cognitive-Behavior Therapy and Research, National Center of Neurology and Psychiatry, Hsinchu, Miaoli, Taiwan

**Keywords:** suicidal ideation, post-traumatic stress disorder, trauma-related guilt, PTSD, suicide

## Abstract

**Background:**

As a mental health issue, suicide is a growing global concern, with patients who have post-traumatic stress disorder (PTSD) being at particularly high risk. This study aimed to investigate whether the link between PTSD and suicidal ideation is mediated by trauma-related guilt.

**Methods:**

Data were obtained from Wave 1, Time 1 (November 2016), and Time 2 (March 2017) of the National Survey for Stress and Health (NSSH) in Japan. The NSSH is an online longitudinal survey conducted on Japan’s national population aged 18 years and older. The cumulative response rate of the survey was 66.7% at Time 2. A total of 1,005 patients with PTSD were included for analyses. The severity of PTSD symptoms was assessed with PTSD DSM-5 Checklist, and the trauma-related guilt were assessed using the two subscales (hindsight-bias/responsibility and global guilt scale) of the trauma-related guilt inventory (TRGI). Suicidal ideation was evaluated using the suicidal ideation attributes scale (SIDAS). Pearson’s correlation was used to investigate the associations among PTSD symptoms, TRGI scores, and SIDAS scores. Causal mediation analysis was applied to evaluate the causal relationship between PTSD, trauma-related guilt, and suicidal ideation.

**Results:**

Pearson’s correlation did not show patients’ age, gender, and household income significantly associated with SIDAS scores. On the other hand, severities of PTSD symptoms (*r* = 0.361, *p* < 0.001) and trauma-related guilt (*r* = 0.235, *p* < 0.001) were positively associated with SIDAS scores. After adjusting for age, gender, and household income, the mediation analysis revealed that trauma-related guilt significantly mediates the effects of PTSD symptoms on suicidal ideation.

**Conclusion:**

Our results implied that trauma-related guilt may represent a critical link between PTSD and suicidal ideation, which may be a noteworthy target for therapeutic intervention.

## Introduction

Post-traumatic stress disorder (PTSD) is a mental health condition that involves intense and prolonged fear or anxiety responses after experiencing a traumatic event (1). It affects about 10–40% of people who have been exposed to trauma (2). According to a recent study by the World Health Organization that was conducted in 24 countries, the lifetime prevalence of PTSD varies from 2.1% in low-to-middle-income countries to 5.0% in high-income countries (3). Symptoms of PTSD include flashbacks, hyperarousal, hypervigilance, intrusive thoughts with emotional responses, nightmares, negative thoughts or feelings that began or worsened after the trauma, or avoidance of situations that may trigger memories of traumatic events (1). PTSD patients commonly have other psychiatric disorders. Based on the data from epidemiologic surveys, the most common psychiatric comorbid disorders are depression, substance use disorders, and other anxiety disorders (4). Moreover, PTSD is also linked to chronic poor physical health, such as asthma (5), rheumatic disorder, eczema (5), arthritis (5), musculoskeletal pain disorders, metabolic syndrome (6), fibromyalgia (7), sleep disorders (8), and cardiovascular disorder (9), which leads to poor quality of life. Both mental and physical comorbidities may increase the risk of suicide in PTSD patients (10). Indeed, emerging evidence has shown that patients with PTSD are at an increased risk of suicidal behavior (11). In a nationwide population-based study in Sweden consisting of 3.1 million people, individuals diagnosed with PTSD were twice as likely to die by suicide (12). Another two population-based studies, both conducted in registered data in Danish population, have examined the association between PTSD and death by suicide. The first study was a case–control investigation of suicide deaths in Denmark from 1994 to 2006, which found that those with a diagnosis of PTSD had 5.3 times higher odds of suicide compared to those without the diagnosis (13). In a second study by the same research team, which examined a Danish population cohort from 1995 to 2011, suicide rates were found to be 13 times higher among individuals diagnosed with PTSD (14). Furthermore, a meta-analysis demonstrated a strong association between PTSD and increased suicidality, including suicidal ideation, plans, attempts, behaviors, and completed suicides (15). Our recent research works have demonstrated a positive relationship between specific PTSD symptoms and suicidal ideation (1, 16, 17). Our results have demonstrated that re-experience feelings and negative alterations in cognition and mood symptoms associated with the trauma were significantly associated with increased suicidal ideation in PTSD patients (1, 17).

In addition, emotional responses to trauma may play a crucial role in PTSD symptom severity and may contribute to an increased risk of suicidal ideation (18). One possible explanation is that PTSD increases suicidal ideation through related negative cognitions about the meaning of the trauma (19), such as negative cognitions about self, the world, and self-blame. In a sample of active duty military personnel, guilt predicted greater suicidal ideation after adjusting for the effects of PTSD and depression (20), indicating that PTSD increase the risk of suicidal ideation *via* cognitive-affective processes that are more proximally related to SI other than PTSD.

Guilt is conceptualized as a psychological response linked to a specific behavior (e.g., feel bad about what I have done) and is referred to behavior-related negative self-conscious emotion and the feeling of guilt often leads to the development of sense of remorse feelings (20).

Previous studies have shown that feelings of guilt after a traumatic event may lead to increased PTSD symptomatology, suggesting that guilt in reaction to trauma may be part of the causal mechanism leading to the development of PTSD. Specifically, trauma-related guilt is strongly correlated with re-experiencing PTSD symptoms (21), which may contribute to suicidal ideation (22). For instance, Hendin et al. have found that combat-related guilt was the most significant predictor of suicide attempts and SI in a clinical sample of Vietnamese combat veterans (23). In addition, McLean et al.’s research also showed that combat-related guilt was significantly correlated with the endorsement of suicidal ideation in a clinical sample of Iraq and Afghanistan combat veterans (19). Furthermore, in 69 active duty military personnel, Bryan et al. found that guilt has a particularly strong relationship with suicidal ideation (20). However, these studies are limited by conducting in military personnel, not in the general population, not measuring guilt and suicidal ideation with validated tools, and cross-sectional design. Moreover, these studies mostly adopted linear or logistic regression to analyze the relationship between guilt and suicidal ideation. In clinical studies, intermediate variables are usually collected, but they are often incorrectly treated as confounding factors (24).

Thus, these intermediate variables (e.g., comorbid mental health conditions) are usually inappropriately adjusted on the causal pathway between PTSD and suicide in multivariable linear or logistic regression models depending on the types of outcome variable, which fails to disentangle the underlying mediating processes (24). Therefore, the exact cause-and-effect relationship between PTSD, trauma-related guilt, and suicidal ideation in previous studies could not be fully addressed (25).

Causal mediation analysis has been of increasing interest in mental health research as a methodology to examine the exact mechanisms by which an exposure leads to an outcome. Research examining the exact causal relationship among trauma-related guilt, PTSD symptoms, and suicidal ideation is relatively lacking. A better understanding of the role of guilt in the associations between PTSD and suicidal ideation is important, as there is a crucial need to identify suicide risk factors and hence targeted therapeutic interventions can be implemented. Therefore, the aim of this study was to investigate the causal relationship between PTSD symptoms, trauma-related guilt and suicidal ideation in a longitudinal follow-up Japanese population. In the present study, we hypothesize that guilt mediates the relationship between PTSD symptom and suicidal ideation.

## Materials and methods

### Database

The data we used in the present study were extracted from the National Survey for Stress and Health (NSSH) which was an online survey conducted nationwide between 2016 and 2017. Detailed information for the NSSH can be found in our previous publications (1, 16, 17, 26). Briefly, NSSH consisted of two surveys: Wave 1 and Wave 2. Wave 1 (*n* = 3,090) consisted of the screening (November 2016), Time 1 (November 2016), and Time 2 surveys (March 2017). Wave 2 (*n* = 3,090) consisted of screening and a Time 1 survey (both in March 2017). Our study was conducted in two waves. Wave 1, which included screening, Time 1, and Time 2 surveys, consisted of 3,090 participants and took place in November 2016, with the Time 2 survey being conducted in March 2017. Wave 2, also with 3,090 participants, took place in March 2017 and included screening and the Time 1 survey. We recruited participants for Wave 1 by sending recruitment emails to 100,077 panelists in November, with the goal of obtaining a sample size of 6,000 individuals. This sample was to include 3,000 patients who met probable diagnostic criteria for PTSD based on the DSM-5 using the PCL-5, 1,000 non-clinical responders who had experienced trauma in the past, and 2,000 non-clinical or subclinical responders who had also experienced trauma. Screening was terminated when we reached half of our target sample size (i.e., 3,000 participants), and participants were asked to complete questionnaires measuring their psychiatric symptoms and psychological processes at Times 1 and 2. Only Wave 1 participants were invited to participate in the Time 2 survey, which took place 4 months after Time 1. We used the longitudinal data collected from Wave 1 in the present study (*n* = 3,090) and the cumulative response rate at Time 2 (*n* = 2,167) was 66.7%. The sample in Wave 1 consisted of subjects with PTSD (*n* = 1,545), subjects experiencing trauma without PTSD (*n* = 930), healthy individuals (*n* = 515), subjects with acute stress disorder (ASD) (*n* = 44), and those experienced trauma within 1 month without ASD symptoms (*n* = 56). The survey participants answered questionnaires evaluating their basic personal data, psychiatric symptoms, and psychological processes at Time 1 and 2. Before responding to the questionnaires, all participants were given a complete description of the research project and gave informed consent. Nine clinical psychologists reviewed the survey contents to evaluate their logical flow, design, validity, and error screening. To improve data quality acquired from the online survey, the computer system automatically excluded respondents who too rapidly answered the questions. The survey was designed to prevent participants from proceeding if some items were unanswered, so no data were missing (except for income). The National Center of Neurology and Psychiatry’s Institutional Review Board approved this study (approval number: A2015-086).

### Participants

This study used data from PTSD patients who were followed up at time 2 (*N* = 1,005). Their data including Time 1 survey data and self-reported suicide ideation at Time 2 were used for analysis.

### Measures

#### Demographic information

Demographic information collected from the subjects included age, gender, and household income.

#### PTSD symptom severity

To assess PTSD symptoms in participants, we used the Japanese version of the 20-item PCL-5, which is available from the National Center for PTSD (26). Respondents answered each item on a 5-point Likert scale (0 = not at all, 1 = a little bit, 2 = moderately, 3 = quite a bit, 4 = extremely), with the 20 items corresponding to DSM-5 diagnostic items. Total PCL-5 scores were used as an indicator of PTSD symptom severity for statistical analysis.

#### Trauma-related guilt

The Trauma-Related Guilt Inventory (TRGI) was designed by Kubany to evaluate the cognitive and emotional aspects of guilt that arise from a traumatic event (27). The TRGI contains 32 items that are divided into three scales: the Distress Scale (six items), the Global Guilt Scale (four items), and the Guilt Cognition Scale (which includes three subscales derived empirically: hindsight-bias/responsibility [seven items], insufficient justification [four items], and wrongdoing [five items], along with six additional general cognition items). Respondents answered all 32 items using a 5-point scale, ranging from “not at all true/never true” to “extremely true/always true” (eight items were reverse-scored). In our online survey, we selected the two subscales of the TRGI (global guilt and hindsight-bias) for data analysis, using scores from these subscales as indicators of the severity of trauma-related guilt in NSSH.

#### Suicidal ideation

The severity of suicidal ideation over the previous month was assessed using the Suicidal Ideation Attributes Scale (SIDAS) (28). This scale includes items that evaluate five dimensions of suicidal ideation: frequency of suicidal thoughts, proximity to suicidal acts, level of control over suicidal behavior, degree of distress associated with suicidal thoughts, and impact of suicidal ideation on daily activities. Responses were recorded on an 11-point Likert scale, with higher scores indicating a greater severity of suicidal ideation. For the analysis, the SIDAS scores collected at Time 2 were utilized as an indicator of suicidality.

### Statistical analyses

Kolmogorov–Smirnov test was used to confirm the normal distribution of the data.

Patients with missing household income data were not included in the mediation analysis (*n* = 46). Finally, 959 patients were included in the analysis. Firstly, we examined the associations between patients’ characteristics, PTSD symptoms at Time 1, trauma-related guilt at Time1, and suicidal ideation at Time2 with Pearson’s correlational analysis. Secondly, we conducted a mediation analysis to assess the mediation effect of guilt on the association between PTSD symptoms and subsequent suicidal ideation. Confidence intervals were estimated using 1,000 bootstrap resampling. Potential confounding factors, including age, sex, and household income, were adjusted. The total effect estimates the association between PTSD and SI can be divided into direct and indirect effects. The direct effect of PTSD on suicidal ideation was not transmitted through the mediator (trauma-related guilt). The indirect effect is the path of PSTD to suicidal ideation through the trauma-related guilt. The percentage mediated is the ratio of the total effect to the indirect effect, which estimates the extent to which the total effect pathway affects the mediators. Based on *the central limit theorem*, the TRGI, PCL-5, and SIDAS scores were assumed to be normally distributed. All statistical analyses were conducted using SAS version 9.4 (SAS Institute Inc., Cary, NC, United States). PROC CAUSALMED was used for the mediation analyses. Statistical significance was assessed using 95% confidence intervals or a *p value* < 0.05.

## Results

### Characteristics of study participants

The sample (*N* = 1,005) consisted of 500 men (49.8%) and 505 women (50.1%). The participants’ mean age was 44.1 years [standard deviation (SD) = 9.5]. Moreover, the mean PCL-5 score was 42.6 (SD = 19.6), the mean TRGI-score was 32.9 (SD = 12.2), and the mean SIDAS score was 22.0 (SD = 10.2) (Table 1).

**Table 1:** Characteristics of included study participants.

	PTSD subjects (*N* = 1,005)
Age (mean, SD)	(44.1, 9.5)
Gender (male/female)	(500/505)
Household income (yen)^a^	
0–1,999,999	194
2,000,000–3,999,999	224
4,000,000–5,999,999	183
6,000,000–7,999,999	104
8,000,000–9,999,999	71
Over 10,000,000	63
PCL-5 scores (mean, SD)	(42.6, 19.6)
SIDAS scores (mean, SD)	(22.0, 10.2)
TRGI scores (mean, SD)	(32.9; 12.2)

^a^46 missing data for household income.

### Correlational analyses

Results of Pearson’s correlation did not show patients’ age, gender, and household income significantly associated with SIDAS scores (Table 2). On the other hand, severities of PTSD symptoms (*r* = 0.361, *p* < 0.001) and trauma-related guilt (*r* = 0.235, *p* < 0.001) were significantly associated with SIDAS scores. In addition, older age was significantly associated with lower PCL-5 scores (*r* = −0.089, *p* < 0.05) and TRG scores (*r* = −0.099, *p* < 0.05).

**Table 2:** Results of Pearson’s correlation analyses among variables.

	Sex	Age	PCL-5	SIDAS	TRG	Income
Sex	1					
Age	−0.333^**^	1				
PCL	0.008	−0.089^*^	1			
SIDAS	−0.034	−0.016	0.361^***^	1		
TRG	0.043	−0.099^*^	0.473^***^	0.235^***^	1	
Income	−0.019	0.021	−0.042	−0.002	−0.038	1

^*^p < 0.05,^**^p < 0.001, ^***^p < 0.0001. PCL-5, Post-traumatic Stress Disorder Checklist for DSM-5; SIDAS, suicidal ideation attributes scale; TRG, trauma-related guilt.

### Causal mediation analyses

**Figure 1:**
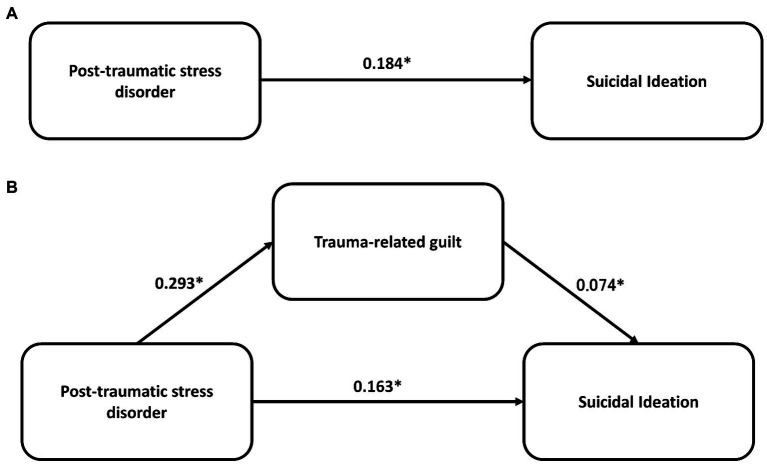
The mediation effect of trauma-related guilt on the association between post-traumatic stress disorder and suicidal ideation. *p < 0.001.

Table 3 shows the results of the mediation effect of trauma-related guilt between baseline PTSD symptoms and suicidal ideation adjusted for age, sex, and household income. There was a significant total effect of PSTD on suicidal ideation (coeff. = 0.184, *p* < 0.001). The direct (coeff. =0.160, *p* < 0.001) and indirect effects (coeff. = 0.024, *p* = 0.006) of PSTD on suicidal ideation were both significant, respectively. The proportion effect mediated by guilt was 12.7%. The standardized coefficients for the direct and indirect effects of the path analysis are shown in Figure 1.

**Table 3:** Summary of coefficient of mediation analysis among PTSD severity, trauma related guilt, and suicide ideation.

Summary of effects of mediation analysis						
	Estimate (coefficient)	Standard Error	Wald 95% Confidence Limits		*Z*	Pr > |Z|
Total Effect	0.184	0.01573	0.1528	0.2145	11.67	**<0.0001**
Natural Direct Effect	0.160	0.01776	0.1255	0.1951	9.02	**<0.0001**
Natural Indirect Effect	0.023	0.00848	0.006758	0.04	2.76	**0.0058**
Percentage Mediated by TRG	12.730	4.7287	3.462	21.9983	2.69	**0.0071**

PTSD, post-traumatic stress disorder; TRG, trauma-related guilt; Significant results were marked with bold characters (*p* < 0.05).

## Discussion

To our knowledge, this is the first study to investigate the mediating role of trauma-related guilt on the link between PTSD symptoms and suicidal ideation in a longitudinally followed population using causal mediation analysis. We found that trauma-related guilt significantly mediated the association between PTSD symptoms and suicidal ideation. Our findings may suggest that trauma-related guilt is a highly relevant component of PTSD, and substantially contributes to the development of suicidal ideation.

Our findings that guilt was positively correlated with suicidal ideation are similar to those reported in previous studies (20, 23). Using generalized linear regression to analyzed the associations between guilt, PTSD symptoms, and suicidal ideation, Bryan et al. concluded that guilt, measured by the Harder Personal Feelings Questionnaire (29), fully mediated the relationship between depression and PTSD symptom severity with SI in 69-nine active duty military personnel. Hendin et al.’s research showed that combat guilt was the most significant predictor of both suicide attempts and preoccupation with suicide in 100 veterans with PTSD diagnosis (23). Among 366 treatment-seeking military personnel with PTSD, McLean et al. used structural equation modeling to examine the associations among combat exposure, PTSD severity, social support, depressive symptoms, guilt, and trauma-related guilt on suicidal ideation. They found that trauma-related guilt plays an important role in suicidal ideation. However, due to differences in study population and statistical methods adopted in these researches, it is difficult to make direct comparison between their study results and ours.

Previous studies have identified trauma-related guilt as a risk factor for developing post-traumatic psychopathology and have demonstrated ongoing guilt to trauma with poorer treatment outcomes (30–32). The results of these studies suggest that therapeutic interventions targeting guilt related to trauma may help to alleviate symptoms of PTSD and improve patients’ clinical outcomes. However, current therapeutic interventions primarily focus on alleviating fear in PTSD patients (33). Advancements in understanding cognitive-affective processes that underlie PTSD emphasize the possible benefits of more individualized treatment approaches that target guilt-related feelings and thoughts associated with the traumatic event (34). Based on the present data, reducing guilt among individuals with PTSD may reduce their risk of suicide. PTSD treatment and suicide risk management may be enhanced by assessing and directly targeting guilt in the intervention plan if identified. Such interventions may be especially beneficial to trauma groups that experience high personal involvement and, therefore, greater levels of guilt and more severe PTSD symptomatology (35).

Our findings have important clinical implications, suggesting that clinicians should consider assessing and addressing trauma-related guilt in patients with PTSD, as this may improve risk assessment and treatment outcomes. Previous studies have demonstrated that pharmacological interventions, such as sertraline, have been effective in reducing guilt in combat veterans with PTSD symptoms (36). Furthermore, various psychological interventions, including prolonged exposure therapy (36), cognitive processing therapy (37), and trauma-informed guilt reduction therapy (38), have shown promising results in helping individuals’ process trauma-related guilt and reducing the severity of PTSD symptoms. Therefore, clinicians should consider these approaches when treating patients with PTSD who experience trauma-related guilt.

In a systematic review of functional neuroimaging studies, the underlying neural correlates of guilt has been shown to be associated with dysfunctions in prefrontal cortex, including the dorsomedial prefrontal cortex (DMPFC), the ventrolateral prefrontal cortex (VLPFC), and the dorsolateral prefrontal cortex (DLPFC) (39). These brain areas overlapped with those shown to be dysfunctional in PTSD patients in functional neural imaging studies (40). Specifically, PTSD is associated with dysfunction in three large-scale functional networks in the brain: the executive control network (ECN), the default mode network (DMN), and salience network (SN) (41). The DLPFC plays an important role in the ECN that is involved in executive functioning, working memory, and emotional regulation (42). DMPFC is crucial in the DMN related to self-referential processing and episodic memory (41). More recently, non-invasive brain stimulation (NIBS) such as repetitive transcranial magnetic stimulation (rTMS) or transcranial direct current stimulation (tDCS) has been shown to alleviate PTSD-associated symptoms (43). For instance, high frequency stimulation on the right DLPFC has been shown to reduce re-experiencing feelings, avoidance, hyperarousal (44), related depressive (44, 45) and anxiety symptoms (44), and suicidal ideation (46). Moreover, tDCS has also been demonstrated to alleviate PTSD symptoms by stimulating the DLPFC or ventromedial PFC (43). Whether NIBS alleviate trauma-related guilt in PTSD patients remains unknown, and future studies are warranted.

## Limitations

The present study has several limitations. To start with, we utilized an online self-report questionnaire to evaluate the risk of suicide and its related clinical and functional factors. While participation in the study was optional and confidential, previous research suggests that anonymous online surveys may elicit a higher rate of sensitive responses (47). Secondly, it should be noted that our findings are specific to online assessments and may not apply to paper-based assessments or face-to-face interviews. Nonetheless, the psychometric data obtained in this study may inform the development of online epidemiological surveys or telemedicine practices in the future. Thirdly, since our sample only included adults aged 18 years and older, caution should be exercised when extrapolating our results to the child and adolescent populations. Fourth, we cannot fully exclude the possibility of interactions between these variables in the mediation analysis. Fifth, we did not evaluate the guilt symptoms in time 2 point, given recent findings suggesting that some PTSD patients may have resilience after trauma overtime (1, 16). Finally, the study participants were restricted to individuals who have online access and have registered as survey panelists. It is uncertain whether these findings can be extended to other data collection methods.

## Conclusion

The significance of TRG in suicidal ideation among patients with PTSD is emphasized by our study. Additionally, the study underscores the importance of paying more attention to TRG in both research and clinical practice involving individuals with PTSD and SI.

## Data availability statement

The raw data supporting the conclusions of this article will be made available by the authors, without undue reservation.

## Ethics statement

The studies involving human participants were reviewed and approved by Institutional review board of the National Center of Neurology and Psychiatry (approval number: A2015-086). The patients/participants provided their written informed consent to participate in this study.

## Author contributions

C-SW analyzed the data. P-HC and S-CW drafted the manuscript. MI conceived and designed the study and managed study administration, and including the ethical review process. All authors provided critical comments on the manuscript related to intellectual content, contributed to the article, and approved the submitted version.

## Funding

This study was supported by a Grant-in-Aid for Scientific Research (A) (15H01979), awarded to MI, from the Japan Society for the Promotion of Science, Tokyo, Japan.

## Conflict of interest

The authors declare that the research was conducted in the absence of any commercial or financial relationships that could be construed as a potential conflict of interest.

## Publisher’s note

All claims expressed in this article are solely those of the authors and do not necessarily represent those of their affiliated organizations, or those of the publisher, the editors and the reviewers. Any product that may be evaluated in this article, or claim that may be made by its manufacturer, is not guaranteed or endorsed by the publisher.
